# Supplementary material for: Effect of Supplementing a Bacillus Multi-Strain Probiotic to a Post-Weaning Diet on Nutrient Utilisation and Nitrogen Retention of Piglets
Source: Animals (Basel). 2023 Nov 21;13(23):3597. doi: 10.3390/ani13233597 (PMC10705099; doi:10.3390/ani13233597)
Supplement: Supplementary file 1 [file animals-13-03597-s001.zip › animals-2713525-supplementary.pdf]

animals

MDPI

Supplementary Material

Effect of supplementing a *Bacillus* multi-strain probiotic to a post-weaning diet on nutrient utilization and nitrogen retention of piglets.

Anne M.S. Huting, L. Vanessa Lagos, Lea H.B. Hansen, and Francesc Molist

Supplementary Table S1. Analysed dietary nutrient content of the experimental diets (i.e. control diet = CD; supplemented diet = SD), in g/kg.

Treatment	Moisture	Ash	CP	CFat	CF	Starch	Sugar	Ti
CD	112	55	186	51	37	370	40	2.95
SD	109	56	188	51	37	373	43	3.01

Supplementary Table S2. CFU recovery analysis of the experimental diets (i.e. control diet = CD; supplemented diet = SD).

Treatment	Sample type	Expected, CFU/g	Result, CFU/g
CD	Mash	<1.00E+05	3.10E+04
CD	Pellet	<1.00E+05	3.10E+04
SD	Mash	1.10E+06	1.51E+06
SD	Pellet	1.10E+06	1.41E+06

Supplementary Table S3. The effect of experimental treatment (i.e. control diet = CD; supplemented diet = SD) on piglet performance.

Parameter[1]	CD	SD	SEM	*P*-value
Weaning age, day				
Day 0[2]	29.8	29.6	0.53	0.64
Body weight, kg				
Day 0[2]	8.48	8.48	0.013	1.00
Day 11	11.5	11.2	0.26	0.46
Day 19[2]	15.3	15.0	0.62	0.74
ADG, g/piglet				
Day 0-11	275	246	23.5	0.46
Day 11-19[2]	285	263	11.2	0.27
ADFI, g/piglet				
Day 0-11	285	263	11.2	0.27
FCR, g/g				
Day 0-11	1.05	1.09	0.088	0.78
Faecal score[2]				
Day 0-11	6.78	6.96	0.239	0.63
Day 11-19[2]	6.69	6.72	0.105	0.86

[1] The experimental unit was pen (*n* = 4) in case not stated otherwise. Replicate (1 to 4) was used as random effect. The experimental results were analysed using a two-way analysis of variance (ANOVA) by GenStat®.
[2] An 8-point scale from severe water thin diarrhoea to hard, dry and lumpy faeces was used for faecal consistency determination. Faecal score 6 was considered the optimal faecal score (see for protocol [13]).
[3] The experimental unit was piglet (*n* = 8). Replicate (1 to 8) was used as random effect. The experimental results were analysed using a two-way analysis of variance (ANOVA) by GenStat®.

Supplementary Table S4. The effect of experimental treatment (i.e. control diet = CD; supplemented diet = SD) on apparent ileal digestibility of amino acids.[1]

Parameter	CD	SD	SEM	*P*-value
Indispensable AA, %				
Arg	77.6	76.9	2.47	0.87
His	78.0	77.7	2.34	0.93
Ile	74.2	75.2	2.53	0.80
Leu	75.6	76.5	2.29	0.80
Lys	82.3	83.7	1.97	0.64
Met[2]	88.1	88.5	-	0.85
	(-93.6 - 93.8)	(-93.5 - 93.8)		
Phe	78.8	78.4	2.52	0.93
Thr[2]	74.9	75.5	-	0.85
	(-84.9 - 85.7)	(-84.8 - 85.8)		
Trp	71.6	72.3	2.28	0.84
Val	75.6	78.0	3.20	0.61
Dispensable AA, %				
Ala	65.4	64.9	3.48	0.92
Asp	69.7	68.2	3.53	0.78
Cys	66.0	61.6	4.39	0.51
Glu	81.0	79.2	2.73	0.65
Gly	47.6	44.6	6.95	0.77
Pro	71.4	56.6	8.43	0.27
Ser	73.1	71.6	2.48	0.69
Tyr	70.5	73.4	3.06	0.54
All indispensable AA, %	77.0	77.6	2.45	0.88
All dispensable AA, %	72.4	67.0	4.17	0.40
All AA, %	74.5	71.7	3.34	0.60

[1] The experimental unit was piglet (n = 8). Replicate (1 to 8) was used as random effect. The experimental results were analysed using a two-way analysis of variance (ANOVA) by GenStat®. From replicate 2 of treatment SD not enough ileal content was left to perform the AA analysis, therefor this piglet was treated as missing value.

[2] This parameter was considered not normally distributed in its original form (i.e. Shapiro Wilk P<0.05). Transformation suggestions were made by the "ABOXCOX" procedure in Genstat. The apparent ileal Met and apparent ileal Thr digestibility coefficients were transformed using (X-100)^-4. For presentation purposes the calculated means were back transformed and are presented together with the 95% confidence interval (CI, using Bonferroni inequality) instead of SEM.

Supplementary Table S5. The effect of experimental treatment (i.e. control diet = CD; supplemented diet = SD) on pH of the digesta content.

Parameter[1]	CD	SD	SEM	*P*-value
Jejunum	6.50	6.48	0.085	0.86
Ileum	6.70	6.63	0.095	0.64
Colon	5.75	5.84	0.037	0.15

[1] The experimental unit was piglet (n = 8). Replicate (1 to 8) was used as random effect. The experimental results were analysed using a two-way analysis of variance (ANOVA) by GenStat®. Piglets from replicate 2 (i.e. one piglet from the CD and one piglet from the SD treatment) had a ileum pH content that was > 2.5 times lower than the standard error of the residuals and were therefore considered outlier.
